# Diagnostic Yield, Clinical Predictors and Histopathologic Findings in Pediatric Colonoscopy

**DOI:** 10.3390/jcm15218520

**Published:** 2026-11-02

**Authors:** Nazan Öztürk Akgümüş, Kamile Merve Bircan, Mustafa Akçam

**Affiliations:** 1 Department of Pediatrics, Sakarya Yenikent State Hospital, Sakarya 54100, Türkiye; nazanozturk20@hotmail.com; 2 Division of Pediatric Gastroenterology, Hepatology and Nutrition, Department of Pediatrics, Faculty of Medicine, Suleyman Demirel University, Isparta 32260, Türkiye; makcam32@gmail.com

**Keywords:** pediatric colonoscopy, diagnostic yield, histopathology, abdominal pain, rectal bleeding, chronic diarrhea

## Abstract

**Objective:** Pediatric colonoscopy is widely used for the evaluation of gastrointestinal symptoms, but its diagnostic yield varies according to clinical presentation. This study aimed to assess the endoscopic and histopathologic diagnostic yield of pediatric colonoscopy, identify clinical predictors of positive findings, and evaluate the additional value of biopsy in macroscopically normal findings. **Methods:** This retrospective single-center study included children aged ≤18 years who underwent colonoscopy at a tertiary pediatric gastroenterology unit. Among 250 colonoscopy records, 230 index colonoscopies were included in patient-level analyses. A specific histopathologic diagnosis included Crohn disease, ulcerative colitis, eosinophilic colitis, infectious colitis, juvenile polyp, or adenomatous polyp. Multivariable logistic regression was used to identify independent predictors of abnormal endoscopic findings and specific histopathology. **Results:** The mean age was 12.0 ± 4.7 years, and 52.2% of patients were female. Abnormal endoscopic findings were identified in 155/230 patients (67.4%). Biopsies were obtained in 201 patients, of whom 58 (28.9%) had a specific histopathologic diagnosis. Children with alarm features had higher endoscopic yield than those with isolated abdominal pain (73.8% vs. 45.8%; OR 3.33; *p* < 0.001) and higher specific histopathologic yield among biopsied patients (34.9% vs. 8.2%; OR 6.04; *p* < 0.001). Rectal bleeding independently predicted abnormal endoscopy (aOR 4.33, 95% CI 1.98–9.48; *p* < 0.001). Younger age (aOR 0.90 per year, 95% CI 0.84–0.97; *p* = 0.008), rectal bleeding (aOR 4.61, 95% CI 2.12–10.01; *p* < 0.001), and chronic diarrhea (aOR 2.37, 95% CI 1.15–4.85; *p* = 0.019) independently predicted specific histopathology. Only 1/62 patients (1.6%) with a macroscopically normal colonoscopy had a specific histopathologic diagnosis. **Conclusions:** Pediatric colonoscopy has substantially higher diagnostic yield in children with alarm features, particularly rectal bleeding and chronic diarrhea, whereas isolated abdominal pain is associated with low disease-specific histopathologic yield. Clinical presentation and age may help improve patient selection for colonoscopy.

## 1. Introduction

Colonoscopy is an essential diagnostic and therapeutic procedure in pediatric gastroenterology, allowing direct visualization of the colonic and terminal ileal mucosa, histopathologic sampling, and therapeutic intervention when required. Its use has expanded with increasing recognition of inflammatory bowel disease (IBD) and other gastrointestinal disorders in children. Common indications include rectal bleeding, chronic abdominal pain, chronic diarrhea, iron deficiency anemia, growth impairment, suspected IBD, and colonic polyps [1,2,3,4]. Because colonoscopy requires bowel preparation and usually sedation or anesthesia, appropriate patient selection is particularly important in pediatric practice.

The diagnostic yield of pediatric colonoscopy is not uniform across indications. Contemporary pediatric studies demonstrate that the probability of obtaining clinically meaningful findings varies according to the presenting symptoms and preprocedural suspicion of organic disease [1,2,3]. Rectal bleeding, chronic diarrhea, iron deficiency, weight loss, growth impairment, and suspected IBD have generally been associated with greater diagnostic yield, whereas isolated or nonspecific abdominal pain is more frequently associated with normal or nonspecific findings [1,2,3,5]. In children with recurrent abdominal pain, the presence of additional alarm features may therefore provide an important basis for deciding whether invasive investigation is warranted [5]. Recent evidence also suggests that laboratory and clinical variables may complement symptom-based assessment when estimating the probability of an abnormal colonoscopy [6].

Age may further influence both the indication for colonoscopy and the expected diagnostic spectrum. Preschool children have a distinct profile, with rectal bleeding being a frequent indication and juvenile polyps representing an important positive finding [7]. In contrast, IBD constitutes an increasingly important diagnosis in older children and adolescents [2,8]. Consequently, the interpretation of symptoms and expected diagnostic yield should take the child’s age into consideration.

The diagnostic value of colonoscopy also depends on procedural quality and appropriate histopathologic assessment. Complete examination, adequate bowel preparation, cecal intubation, and terminal ileal intubation are recognized components of high-quality pediatric ileocolonoscopy [9,10,11]. The Pediatric Endoscopy Quality Improvement Network (PEnQuIN) established pediatric-specific quality standards, including minimum targets for adequate bowel preparation and terminal ileal intubation [9,10]. Histopathologic assessment provides an additional diagnostic dimension because macroscopic and microscopic findings may not always be concordant. Pediatric studies have demonstrated that clinically relevant histologic abnormalities can occasionally be identified despite normal-appearing mucosa, although the benefit of routine biopsy appears to depend strongly on the underlying clinical context [12,13,14].

Although the association between alarm symptoms and abnormal colonoscopic findings has been well established in pediatric populations, the distinction between macroscopic endoscopic abnormalities and disease-specific histopathologic diagnoses remains clinically relevant. Previous studies have evaluated diagnostic yield using varying definitions, which may complicate the interpretation of findings across different clinical indications. Therefore, the present study aimed to evaluate macroscopic endoscopic abnormalities and specific histopathologic diagnoses as separate outcomes within the same pediatric cohort, examine their associations with clinical characteristics and age, and assess the additional disease-specific histopathologic yield of biopsy in macroscopically normal examinations. By distinguishing nonspecific endoscopic abnormalities from disease-specific histopathologic findings, this study seeks to provide a more clinically meaningful interpretation of diagnostic yield rather than to identify previously unrecognized clinical predictors.

## 2. Materials and Methods

### 2.1. Study Design and Population

This retrospective single-center study included patients aged ≤18 years who underwent colonoscopy at the Pediatric Gastroenterology, Hepatology and Nutrition Endoscopy Unit of Suleyman Demirel University Faculty of Medicine between January 2016 and February 2021. Clinical, endoscopic, and histopathologic data were retrospectively obtained from the endoscopy unit archive and hospital medical records. For patients who underwent more than one colonoscopy during the study period, only the first (index) procedure was included in the analysis to ensure that each patient contributed only once to the study cohort. The study was originally conducted in 2021, with data collection completed in February 2021. The decision to prepare the findings for publication was made subsequently. Thus, the study period reflects the original data collection timeframe rather than a deliberate exclusion of more recent procedures.

The study was approved by the Suleyman Demirel University Faculty of Medicine Ethics Committee on 23 March 2021 (no:149). Written informed consent was obtained from parents and, for patients older than 12 years, from the patients themselves.

### 2.2. Data Collection and Definitions

Recorded variables included age, sex, presenting symptoms, colonoscopy indications, bowel preparation quality, terminal ileal intubation, endoscopic findings, biopsy status, and histopathologic findings. Rectal bleeding included hematochezia, melena, and/or positive fecal occult blood testing. The original study also recorded screening and follow-up indications, including previously diagnosed IBD and colorectal polyps.

For the present analysis, predefined alarm features included rectal bleeding, chronic diarrhea, iron-deficiency anemia, weight loss, and growth failure. Patients with abdominal pain without any predefined alarm feature or another screening indication were categorized as having isolated abdominal pain. Patients with other indications who fulfilled neither definition were excluded only from the isolated abdominal pain versus alarm-feature comparison. Bowel preparation quality was categorized as adequate, partially adequate, or inadequate according to the endoscopy report. Terminal ileal intubation was evaluated separately.

### 2.3. Endoscopic and Histopathologic Assessment

Colonoscopy procedures were performed by a pediatric gastroenterologist using a Fujinon EC-T530LP video colonoscope (FUJIFILM Corporation, Tokyo, Japan) under intravenous sedation with midazolam and ketamine. Biopsy specimens were fixed in formalin and evaluated in the institutional pathology laboratory. Biopsy sampling was performed as part of routine clinical care rather than according to a prospectively standardized research protocol. Consequently, the number and distribution of biopsy specimens were not uniform across patients. Histopathologic analyses were restricted to patients from whom biopsy specimens were obtained, and patients without biopsy specimens were not classified as having negative histopathologic findings.

Endoscopic diagnostic yield was operationally defined as the presence of at least one macroscopic abnormality, regardless of its presumed clinical significance or relationship to the presenting symptoms. Accordingly, findings such as hemorrhoids, anal fissures, and nonspecific colitis were included in this broad definition. Examinations without macroscopic abnormalities were classified as normal. To distinguish the detection of any visible abnormality from the identification of disease-specific pathology, specific histopathologic diagnoses were evaluated as a separate outcome. Endoscopic findings included inflammatory bowel disease, nonspecific colitis, polyps, hemorrhoids, anal fissure, terminal ileal nodularity, and other abnormalities. Histopathologic findings were categorized as specific or nonspecific. Disease-defining diagnoses were considered specific histopathologic diagnoses, whereas normal histology and nonspecific inflammatory changes were classified as nonspecific.

### 2.4. Statistical Analysis

Statistical analyses were performed using IBM SPSS Statistics version 23 (IBM Corp., Armonk, NY, USA). Categorical variables were expressed as numbers and percentages, and continuous variables as mean ± standard deviation or median (interquartile range), as appropriate. Categorical variables were compared using the chi-square or Fisher’s exact test, as appropriate. Continuous variables were compared using parametric or nonparametric tests according to their distribution. Multivariable logistic regression analyses were performed to identify independent predictors of abnormal endoscopic findings and specific histopathologic diagnoses. The eight covariates included in the models were age, sex, growth failure, weight loss, rectal bleeding, abdominal pain, chronic diarrhea, and iron deficiency anemia. These variables were selected based on their clinical relevance and availability in the retrospective dataset. No data were missing for the eight covariates included in the regression models; therefore, no patients were excluded from these analyses due to missing covariate data. These analyses were intended to assess independent associations between routinely recorded clinical characteristics and diagnostic outcomes rather than to develop or validate a clinical prediction model. Accordingly, the resulting adjusted odds ratios were interpreted as measures of association and not as components of a clinical prediction score. Results were reported as odds ratios (ORs) with 95% confidence intervals (CIs). A two-sided *p* value < 0.05 was considered statistically significant.

## 3. Results

### 3.1. Study Population, Indications, and Procedural Characteristics

A total of 250 colonoscopy records were identified during the study period. After separating repeat procedures, 230 index colonoscopies were included in the patient-level primary analyses, while 20 additional records represented repeat examinations.

The demographic, clinical, procedural, endoscopic, and histopathologic characteristics of the cohort are summarized in Table 1. The mean age was 12.0 ± 4.7 years and the median age was 13.3 years. Of the 230 patients, 120 (52.2%) were female and 110 (47.8%) were male.

Abdominal pain was the most common indication for colonoscopy (64.3%), followed by rectal bleeding (35.2%) and chronic diarrhea (29.1%). Iron deficiency anemia, weight loss, and growth failure were less frequent. Because patients could have more than one indication, these categories were not mutually exclusive.

Terminal ileal intubation was achieved in 208 patients (90.4%), and 203 examinations (88.3%) were recorded as adequate. Biopsy specimens were obtained in 201 patients (87.4%).

Overall, 155 of 230 index colonoscopies (67.4%) demonstrated at least one abnormal macroscopic finding, whereas 75 (32.6%) were macroscopically normal. The most common endoscopic findings were nonspecific colitis, an appearance compatible with IBD, nodularity, hemorrhoids, anal fissure, and polyps (Table 1).

Values are presented as *n* (%) unless otherwise indicated. Percentages for demographic, clinical, procedural, and endoscopic characteristics were calculated using 230 index colonoscopies as the denominator. Histopathologic percentages were calculated only among the 201 patients who underwent biopsy. Indications and individual endoscopic findings were not necessarily mutually exclusive. Histopathologic categories were also not mutually exclusive, as individual patients could have different findings in biopsy specimens obtained from separate intestinal segments. The 58 patients with a specific histopathologic diagnosis represent unique patients and should not be added to the individual histopathologic categories. IBD, inflammatory bowel disease; SD, standard deviation. Bold text indicates section headings.

Among the 201 biopsied patients, nonspecific histopathologic changes were present in 89 (44.3%), terminal ileal nodular lymphoid hyperplasia in 62 (30.8%), and normal histology in 52 (25.9%). Overall, 58 patients (28.9%) had at least one predefined specific histopathologic diagnosis, most commonly ulcerative colitis and Crohn disease. Each patient was counted only once in the overall specific histopathologic diagnostic yield, regardless of the number of histopathologic findings recorded.

### 3.2. Diagnostic Yield According to Presenting Indication

Endoscopic diagnostic yield differed according to presenting indication. Abnormal macroscopic findings were detected in 86.4% of children with rectal bleeding, 76.5% of those with growth failure, 75.0% of those with iron deficiency anemia, and 73.1% of those with chronic diarrhea. Among children presenting with abdominal pain, the corresponding yield was 59.5%.

### 3.3. Isolated Abdominal Pain Versus Alarm Features

Fifty-nine children had isolated abdominal pain and 160 had at least one predefined alarm feature. The remaining 11 patients had other indications and were not included in this specific comparison. Alarm features were defined as rectal bleeding, chronic diarrhea, growth failure, weight loss, or iron deficiency anemia.

An abnormal endoscopic finding was identified in 27 of 59 children with isolated abdominal pain (45.8%) compared with 118 of 160 children with alarm features (73.8%). Alarm features were therefore associated with more than threefold higher odds of an abnormal endoscopic examination (OR 3.33; *p* < 0.001) (Table 2).

Among patients who underwent biopsy, a specific histopathologic diagnosis was identified in 4 of 49 children with isolated abdominal pain (8.2%) and 51 of 146 children with alarm features (34.9%). Among the four patients with isolated abdominal pain and a specific histopathologic diagnosis, two had ulcerative colitis, one had Crohn disease, and one had a juvenile polyp. The odds of a specific histopathologic diagnosis were approximately sixfold higher in children with alarm features (OR 6.04; *p* < 0.001).

### 3.4. Diagnostic Yield According to Age

Age-specific diagnostic yields are shown in Table 3. Abnormal endoscopic findings were identified in 80.6% of children younger than 6 years, 65.6% of those aged 6–11 years, and 64.7% of adolescents aged 12–18 years. The difference in endoscopic yield across age groups was not statistically significant (*p* = 0.184).

In contrast, specific histopathologic yield differed significantly by age. Among biopsied patients, a specific diagnosis was identified in 17 of 32 children younger than 6 years (53.1%), 12 of 51 children aged 6–11 years (23.5%), and 29 of 118 adolescents aged 12–18 years (24.6%) (*p* = 0.004).

The diagnostic spectrum also varied with age. Infectious and eosinophilic colitis contributed more prominently among younger children, whereas IBD accounted for a greater proportion of specific diagnoses among adolescents.

### 3.5. Histopathologic Findings in Macroscopically Normal Colonoscopies

Among 75 patients with a macroscopically normal colonoscopy, biopsy specimens were obtained in 62. Only one of these patients (1.6%) had a predefined specific histopathologic diagnosis, namely infectious colitis. This patient presented with rectal bleeding, abdominal pain, and chronic diarrhea.

Nonspecific histopathologic abnormalities were recorded in 19 patients and terminal ileal nodular lymphoid hyperplasia in 13, while 39 had normal histology. These findings were not classified as specific histopathologic diagnoses in the primary analysis.

### 3.6. Independent Predictors of Abnormal Endoscopic Findings

Multivariable logistic regression analysis was performed in all 230 index colonoscopies (Table 4). Rectal bleeding was the only independent predictor of an abnormal endoscopic examination (aOR 4.33, 95% CI 1.98–9.48; *p* < 0.001).

Chronic diarrhea showed a trend toward increased endoscopic yield but did not reach statistical significance (aOR 1.83, 95% CI 0.93–3.59; *p* = 0.081). Age, sex, growth failure, weight loss, abdominal pain, and iron deficiency anemia were not independently associated with abnormal endoscopic findings.

### 3.7. Independent Predictors of Specific Histopathologic Diagnosis

The multivariable analysis of specific histopathologic diagnosis was restricted to the 201 patients who underwent biopsy (Table 4).

Increasing age was independently associated with a lower probability of a specific histopathologic diagnosis, with an approximately 10% reduction in odds for each additional year (aOR 0.90, 95% CI 0.84–0.97; *p* = 0.008).

Rectal bleeding was the strongest positive predictor (aOR 4.61, 95% CI 2.12–10.01; *p* < 0.001), while chronic diarrhea was also independently associated with specific histopathologic yield (aOR 2.37, 95% CI 1.15–4.85; *p* = 0.019).

Growth failure showed an increased point estimate but did not reach statistical significance (aOR 3.16, 95% CI 0.88–11.39; *p* = 0.078). Weight loss, abdominal pain, iron deficiency anemia, and sex were not independently associated with a specific histopathologic diagnosis.

## 4. Discussion

The present study evaluated pediatric colonoscopy by distinguishing macroscopic endoscopic abnormalities from disease-specific histopathologic diagnoses. Although the association between alarm symptoms and abnormal colonoscopic findings is well established, our findings highlight the importance of interpreting these two diagnostic outcomes separately. While 67.4% of patients had at least one macroscopic abnormality, a specific histopathologic diagnosis was established in 28.9% of patients who underwent biopsy. Furthermore, specific histopathologic findings were uncommon in biopsies obtained from macroscopically normal examinations (1.6%). Children with predefined alarm features had higher diagnostic yields than those presenting with isolated abdominal pain, with rectal bleeding and chronic diarrhea showing significant associations with specific histopathologic diagnoses. Rather than identifying novel clinical predictors, these findings extend existing evidence by illustrating how diagnostic yield varies according to the outcome definition and clinical presentation. This distinction may contribute to a more nuanced interpretation of pediatric colonoscopy findings.

Importantly, the definition of macroscopic abnormality included findings such as hemorrhoids, anal fissures, and nonspecific colitis, whose clinical relevance may vary according to the presenting symptoms. Consequently, the reported endoscopic yield should not be interpreted as the proportion of patients in whom colonoscopy established a definitive diagnosis.

Direct comparison with previous studies is difficult because definitions of diagnostic yield, patient selection, and inclusion of histopathologic findings vary substantially. Nevertheless, contemporary pediatric series consistently demonstrate that diagnostic yield is strongly influenced by the indication for endoscopy. Fachler et al. reported considerable variation in diagnostic yield across referral indications and emphasized the importance of clinical presentation in selecting children for endoscopy [1]. Similarly, Silva et al. found that ileocolonoscopy had a relatively high diagnostic yield, whereas nonspecific gastrointestinal symptoms required more careful patient selection [3]. Morita et al., examining changing patterns of pediatric diagnostic colonoscopy, reported that abdominal pain had become an increasingly frequent indication, while ulcerative colitis remained a major definitive diagnosis [2]. These studies reinforce the concept that a single overall diagnostic-yield figure should not be interpreted independently of the clinical context in which colonoscopy is performed.

The distinction between isolated abdominal pain and abdominal pain accompanied by alarm features was particularly striking in our cohort. Although 45.8% of children with isolated abdominal pain had some macroscopic abnormality, only 8.2% of biopsied patients in this group had a predefined specific histopathologic diagnosis. In contrast, specific histopathologic abnormalities were identified in 34.9% of biopsied children with at least one alarm feature, corresponding to approximately sixfold higher odds of a specific diagnosis. This distinction is clinically important because recurrent and chronic abdominal pain are common in childhood, and invasive investigation should ideally be directed toward children in whom organic disease is sufficiently likely.

Our findings are consistent with previous pediatric evidence. Singh and Ee found positive histology in only 10% of children undergoing colonoscopy for recurrent abdominal pain and reported no positive histology among children with isolated abdominal pain; rectal bleeding was the only associated indication predicting abnormal histology [5]. In the same study, all children with elevated fecal calprotectin had abnormal histology, suggesting that noninvasive markers may further improve selection for colonoscopy. Fachler et al. likewise demonstrated that diagnostic yield varies according to clinical indication rather than simply the presence of gastrointestinal symptoms [1]. More recently, a pediatric cohort evaluating predictors of abnormal diagnostic colonoscopy identified family history of IBD or hereditary polyposis, lower hemoglobin, and higher C-reactive protein as independent predictors [6]. Taken together, these findings suggest that symptom-based alarm features may be strengthened by laboratory and family-history information when determining pretest probability. Our dataset did not permit incorporation of these variables into the predictive model, but their inclusion represents an important direction for future prospective studies.

Rectal bleeding emerged as the strongest clinical predictor in our study. Children presenting with rectal bleeding had an endoscopic diagnostic yield of 86.4%, and rectal bleeding independently increased the odds of an abnormal endoscopic examination more than fourfold. It was also independently associated with specific histopathology among biopsied patients. This finding is consistent with the broad spectrum of structural and inflammatory conditions presenting with lower gastrointestinal bleeding in children, including polyps, IBD, infectious colitis, and anorectal disease. In preschool children, Bolia et al. identified rectal bleeding as the most frequent indication for colonoscopy and juvenile polyps as the most common positive finding [7]. Thus, unlike nonspecific abdominal pain, visible rectal bleeding appears to provide a strong clinical signal for meaningful lower gastrointestinal pathology.

Chronic diarrhea was independently associated with specific histopathologic diagnosis in our cohort, although its association with macroscopic endoscopic abnormalities did not reach statistical significance. This discrepancy is potentially important. Some disorders presenting with chronic diarrhea may have subtle or absent macroscopic abnormalities but characteristic microscopic changes. Microscopic colitis represents an obvious example: although uncommon in children, diagnosis depends on histopathologic assessment because the mucosa may appear macroscopically normal [15]. The association between chronic diarrhea and specific histopathology in our study therefore supports obtaining appropriate biopsies when persistent diarrhea raises suspicion of mucosal disease, even when gross abnormalities are limited.

Age was another independent determinant of specific histopathologic yield. Children younger than 6 years had the highest yield, with a specific diagnosis identified in 53.1% of biopsied patients, compared with approximately one-quarter of older children. Each additional year of age was associated with an approximately 10% reduction in the adjusted odds of a specific histopathologic diagnosis. Importantly, this should not be interpreted as indicating that colonoscopy is generally more useful in all younger children; rather, it probably reflects differences in indication and disease spectrum among those selected for colonoscopy. Bolia et al. similarly demonstrated a distinct colonoscopic profile among preschool children, with rectal bleeding being common and juvenile polyps representing the most frequent positive finding [7]. In our cohort, infectious and eosinophilic colitis contributed more prominently to specific diagnoses among younger children, whereas IBD accounted for a larger proportion among adolescents. These observations highlight the importance of considering age when estimating both pretest probability and the likely diagnostic spectrum.

The relationship between macroscopic appearance and histopathology represents another important aspect of our findings. Among 75 macroscopically normal colonoscopies, 62 patients underwent biopsy, but only one (1.6%) had a predefined specific histopathologic diagnosis. Glass et al. similarly evaluated grossly normal pediatric colonoscopies and reported 81% agreement between normal endoscopic appearance and normal histology; only 2.5% of grossly normal examinations demonstrated histologic findings consistent with newly diagnosed IBD [12]. Abdominal pain predicted normal histology, whereas established IBD and elevated inflammatory markers were associated with abnormal histology. These findings closely parallel our observation that disease-specific histopathologic yield is very low when the examination is macroscopically normal, particularly in patients with nonspecific symptoms.

However, this finding should not be interpreted as evidence that biopsy can routinely be omitted from normal-appearing mucosa. Scomparin et al. reached a more cautious conclusion in a 10-year pediatric analysis, demonstrating clinically relevant discordance between macroscopic and microscopic findings and reporting that restricting biopsies to visibly abnormal areas could result in missed diagnoses [13]. Earlier pediatric work also demonstrated good overall agreement between endoscopy and histology but identified IBD as an important predictor of abnormal histology despite variation in gross findings [14]. Thus, apparently conflicting evidence is best reconciled by considering pretest probability: routine indiscriminate biopsy may have limited disease-specific yield in low-risk children, whereas systematic histologic sampling remains appropriate when IBD, microscopic colitis, eosinophilic disease, or another mucosal disorder is clinically suspected.

Our definition of specific histopathologic diagnosis is also relevant to interpretation of this finding. Nonspecific inflammatory changes and terminal ileal nodular lymphoid hyperplasia were deliberately excluded from the primary disease-specific outcome. Consequently, the 1.6% yield in macroscopically normal examinations does not indicate that 98.4% of biopsies were microscopically completely normal. Rather, it indicates that only one patient had a histologic finding meeting our predefined criteria for a specific diagnosis. This distinction may partly explain differences between our findings and studies defining any microscopic abnormality as a positive histopathology.

Procedural quality is another important consideration when interpreting diagnostic yield. Terminal ileal intubation was achieved in 90.4% of our index colonoscopies, and 88.3% of examinations were recorded as adequate. PEnQuIN established minimum acceptable targets of ≥85% for terminal ileal intubation, ≥90% for cecal intubation, and ≥80% for adequate bowel preparation in pediatric lower gastrointestinal endoscopy [9,10]. Our terminal ileal intubation and examination adequacy rates therefore compare favorably with these benchmarks. Earlier single-center pediatric data reported a terminal ileal intubation rate of approximately 91% and emphasized the importance of bowel preparation and other modifiable factors in achieving complete examinations [11]. More recent quality-assurance studies continue to demonstrate that procedural experience and practice characteristics can influence terminal ileal intubation and other pediatric colonoscopy quality indicators [16,17]. The high completion rate in our cohort reduces the likelihood that low diagnostic yield in selected clinical groups resulted primarily from technically incomplete examinations.

Our findings have potential implications for clinical decision-making. Rather than considering colonoscopy as a uniform diagnostic test for all children with gastrointestinal complaints, the procedure may be most informative when interpreted within a pretest-probability framework. Rectal bleeding and chronic diarrhea emerged as particularly useful clinical signals in our cohort, whereas isolated abdominal pain was associated with a substantially lower probability of disease-specific histopathology. The addition of noninvasive biomarkers could potentially improve this approach further. Fecal calprotectin, hemoglobin, C-reactive protein, and other inflammatory measures have shown value in identifying children more likely to harbor organic gastrointestinal disease [5,6,18]. Prospective models combining symptoms, growth parameters, family history, and laboratory markers may therefore provide better discrimination than symptom-based selection alone.

The current study has several strengths. First, analyses were performed at the patient level using only the index colonoscopy, thereby avoiding the statistical problem of treating repeated procedures in the same child as independent observations. Second, endoscopic and histopathologic outcomes were analyzed separately, which allowed macroscopic diagnostic yield to be distinguished from disease-specific microscopic diagnoses. Third, histopathologic predictor analyses were restricted to the 201 patients who actually underwent biopsy rather than classifying patients without tissue sampling as histologically negative. Finally, the comparison between isolated abdominal pain and predefined alarm features provides a clinically intuitive framework that may be useful when considering referral for colonoscopy.

Several limitations should be acknowledged. The retrospective, single-center design and tertiary referral setting may introduce selection and information bias and limit generalizability. In addition, the available records did not consistently distinguish colonoscopies performed for initial diagnosis from those performed for follow-up of established conditions, potentially affecting the observed diagnostic yield. Biopsy sampling was not prospectively standardized, and histopathologic analyses were restricted to patients who underwent biopsy.

The multivariable analysis of specific histopathologic diagnoses included 58 outcome events and eight covariates, raising concerns about potential overfitting and estimate instability. Furthermore, important variables, including fecal calprotectin, C-reactive protein, erythrocyte sedimentation rate, family history of inflammatory bowel disease, and detailed growth parameters, were not sufficiently available for adjustment, leaving the possibility of residual confounding. Accordingly, the observed associations should be considered exploratory rather than validated clinical prediction tools.

Finally, data collection ended in February 2021, before the publication of the PEnQuIN quality standards in 2022. Although terminal ileal intubation and examination adequacy rates were comparable with subsequently established benchmarks, this retrospective comparison does not demonstrate adherence to contemporary quality standards. Changes in pediatric endoscopy practice since the study period may further limit the applicability of these findings to current clinical settings.

In conclusion, pediatric colonoscopy yielded substantially fewer disease-specific histopathologic diagnoses than macroscopic abnormalities, underscoring the importance of clearly defining diagnostic outcomes. Rectal bleeding and chronic diarrhea were associated with higher diagnostic yields, consistent with existing pediatric evidence. Although specific histopathologic diagnoses were uncommon in macroscopically normal examinations, this finding should not be interpreted as justification for omitting biopsies when mucosal disease is clinically suspected. Further prospective studies incorporating noninvasive biomarkers are needed to refine patient selection.

## Figures and Tables

**Table 1:** Demographic, clinical, procedural, endoscopic, and histopathologic characteristics of the study population.

Characteristic	*n* (%)
**Demographic characteristics**	
Index colonoscopies	230
Age, years, mean ± SD	12.0 ± 4.7
Median age, years	13.3
Female	120 (52.2)
Male	110 (47.8)
**Major indications**	
Abdominal pain	148 (64.3)
Rectal bleeding	81 (35.2)
Chronic diarrhea	67 (29.1)
Iron deficiency anemia	28 (12.2)
Weight loss	22 (9.6)
Growth failure	17 (7.4)
**Procedural characteristics**	
Terminal ileum reached	208 (90.4)
Adequate examination	203 (88.3)
Partially adequate examination	20 (8.7)
Inadequate examination	7 (3.0)
Biopsy obtained	201 (87.4)
Abnormal endoscopic examination	155 (67.4)
Normal endoscopic examination	75 (32.6)
**Major endoscopic findings**	
Nonspecific colitis	43 (18.7)
Endoscopic impression of IBD	36 (15.7)
Nodularity	36 (15.7)
Hemorrhoid	31 (13.5)
Anal fissure	22 (9.6)
Polyp	18 (7.8)
Skin tag	7 (3.0)
Obstruction	5 (2.2)
Fistula	3 (1.3)
Foreign body	3 (1.3)
Parasitic finding	1 (0.4)
**Histopathologic findings among biopsied patients** (***n* = 201**)	
Nonspecific changes	89 (44.3)
Terminal ileal nodular lymphoid hyperplasia	62 (30.8)
Normal histology	52 (25.9)
Ulcerative colitis	21 (10.4)
Crohn disease	11 (5.5)
Juvenile polyp	8 (4.0)
Infectious colitis	8 (4.0)
Eosinophilic colitis	7 (3.5)
Adenomatous polyp	3 (1.5)
**Any specific histopathologic diagnosis**	**58** (**28.9**)

**Table 2:** Diagnostic yield in children with isolated abdominal pain compared with those with alarm features.

Outcome	Isolated Abdominal Pain	≥1 Alarm Feature	OR	*p*-Value
Abnormal endoscopic finding	27/59 (45.8%)	118/160 (73.8%)	3.33	<0.001
Specific histopathologic diagnosis *	4/49 (8.2%)	51/146 (34.9%)	6.04	<0.001

Alarm features were defined as rectal bleeding, chronic diarrhea, growth failure, weight loss, or iron deficiency anemia. * Histopathologic analysis was restricted to biopsied patients within the two comparison groups; patients with other indications were not included in this comparison. ORs represent the odds associated with ≥1 alarm feature relative to isolated abdominal pain.

**Table 3:** Endoscopic and specific histopathologic diagnostic yield according to age group.

Age Group	Index Colonoscopies, *n*	Abnormal Endoscopy, *n* (%)	Patients Biopsied, *n*	Specific Histopathologic Diagnosis, *n* (%)
<6 years	36	29 (80.6)	32	17 (53.1)
6–11 years	61	40 (65.6)	51	12 (23.5)
12–18 years	133	86 (64.7)	118	29 (24.6)
*p*-value		0.184		**0.004**

Histopathologic percentages were calculated using only patients who underwent biopsy within each age group. Bold values indicate statistical significance (*p* < 0.05).

**Table 4:** Multivariable logistic regression analysis of predictors of diagnostic yield.

Variable	Abnormal Endoscopy aOR (95% CI) *	*p*-Value	Specific Histopathology aOR (95% CI) †	*p*-Value
Age, per year	0.95 (0.89–1.02)	0.155	0.90 (0.84–0.97)	**0.008**
Male sex	1.00 (0.55–1.83)	0.993	1.09 (0.55–2.15)	0.804
Growth failure	1.69 (0.48–5.93)	0.413	3.16 (0.88–11.39)	0.078
Weight loss	1.25 (0.45–3.47)	0.662	2.47 (0.77–7.91)	0.128
Rectal bleeding	4.33 (1.98–9.48)	**<0.001**	4.61 (2.12–10.01)	**<0.001**
Abdominal pain	0.61 (0.29–1.29)	0.198	1.47 (0.66–3.28)	0.344
Chronic diarrhea	1.83 (0.93–3.59)	0.081	2.37 (1.15–4.85)	**0.019**
Iron deficiency anemia	1.21 (0.45–3.29)	0.703	1.34 (0.49–3.69)	0.567

aOR, adjusted odds ratio; CI, confidence interval. * Model based on all 230 index colonoscopies. † Model for specific histopathologic diagnosis restricted to the 201 patients who underwent biopsy. Both models were mutually adjusted for all variables shown. Endoscopic model: *n* = 230 (155 events); specific histopathology model: *n* = 201 (58 events). No missing covariate data. Bold formatting indicates statistically significant *p*-values (*p* < 0.05). An explanation has been added to the table footnote.

## Data Availability

The data supporting the findings of this study are available from the corresponding author upon reasonable request.
